# Computer-Aided Diagnosis Methods for High-Frequency Ultrasound Data Analysis: A Review

**DOI:** 10.3390/s22218326

**Published:** 2022-10-30

**Authors:** Joanna Czajkowska, Martyna Borak

**Affiliations:** Faculty of Biomedical Engineering, Silesian University of Technology, Roosevelta 40, 41-800 Zabrze, Poland

**Keywords:** high-frequency ultrasound, CAD, image classification, image segmentation, image quality assessment, datasets

## Abstract

Over the last few decades, computer-aided diagnosis systems have become a part of clinical practice. They have the potential to assist clinicians in daily diagnostic tasks. The image processing techniques are fast, repeatable, and robust, which helps physicians to detect, classify, segment, and measure various structures. The recent rapid development of computer methods for high-frequency ultrasound image analysis opens up new diagnostic paths in dermatology, allergology, cosmetology, and aesthetic medicine. This paper, being the first in this area, presents a research overview of high-frequency ultrasound image processing techniques, which have the potential to be a part of computer-aided diagnosis systems. The reviewed methods are categorized concerning the application, utilized ultrasound device, and image data-processing type. We present the bridge between diagnostic needs and already developed solutions and discuss their limitations and future directions in high-frequency ultrasound image analysis. A search was conducted of the technical literature from 2005 to September 2022, and in total, 31 studies describing image processing methods were reviewed. The quantitative and qualitative analysis included 39 algorithms, which were selected as the most effective in this field. They were completed by 20 medical papers and define the needs and opportunities for high-frequency ultrasound application and CAD development.

## 1. Introduction

The first computer-aided diagnosis (CAD) systems date back to the mid-1950s and are currently an integral part of daily medical work. The development of CAD systems has been multidirectional, and the scope of the analysis covers data that are one- and multidimensional. Research on the development of dedicated processing techniques and the ease of exchanging experiences and measurement results in many centers worldwide has resulted in computer-aided diagnostics and therapy systems that are commonly used in clinical practice. The best example is radiology, where many years of research into the development of these systems resulted in numerous applications that could support physicians’ work [1]. In addition, the continuous development of imaging techniques is driving research into the development of new methods for analyzing related data.

The fast development of imaging techniques resulting from technological progress opens up new opportunities for the accurate diagnosis of skin diseases and the monitoring of their treatment. Among many other modalities, high-frequency ultrasound (HFUS) indicates new diagnostic paths in skin analysis, enabling the visualization of superficial structures [2,3]. Therefore, it has found plenty of applications in dermatology, allergology, cosmetology, and aesthetic medicine. It is a quantitative tool for measuring skin thickness or the acoustic impedance of different skin layers [4]. At the expense of a lower penetration depth, a higher head frequency improves the spatial resolution of the captured images [5]. This makes surface structures, such as fat and muscle layers, blood vessels, hair follicles, and skin appendages, visible. Moreover, it enables clinicians to easily expose the skin layers, taking into account the epidermis, dermis, and the thick band separating them with reduced intensity (subepidermal low-echogenic band, SLEB), which is characteristic of inflammatory diseases (e.g., eczema, atopic dermatitis, or psoriasis).

Since HFUS is increasingly used in medical practices [6], the demand for automated or semi-automated data analysis in this field is growing. Numerous algorithms dedicated to such processing have already been described in the literature to meet this need. To organize the existing research, we collected all the remarks concerning the published approaches and summarized the opportunities for their application. This is the first survey of computer-aided diagnosis methods for HFUS data. It includes 31 papers that focused on HFUS image processing, which described 39 valuable algorithms that were included in our quantitative and qualitative analysis.

To identify the relevant contributions, PubMed was first queried for papers containing “HFUS” or “High-Frequency Ultrasound” in the title or abstract. This enabled the selection of 20 papers that were the most recent or diversified in terms of HFUS medical applications. Since the review of medical applications was not the goal of our study, the survey briefly summarizes them. They were also an inspiration for the subsequent queries, from which the non-zero results found were “HFUS segmentation”, “epidermis segmentation ultrasound”, “dermis segmentation ultrasound”, “skin lesion segmentation ultrasound”, “highfrequency ultrasound classification”, and “HFUS image quality”.

Since only a few research institutes have worked on HFUS image processing, in our searches we used the authors’ names as queries, “Sciolla B”, “Czajkowska J”, “Marosán P”, or “Kia S”, and their works were reviewed for the HFUS image processing methods.

Finally, we checked the references in the selected papers. Since the HFUS image analysis methods were not widely described in the literature, we did not exclude any papers from the survey. However, the poorly validated methods are not included in the tables summarizing the subsections. The last update to the included papers was made in September 2022.

**Figure 1:**
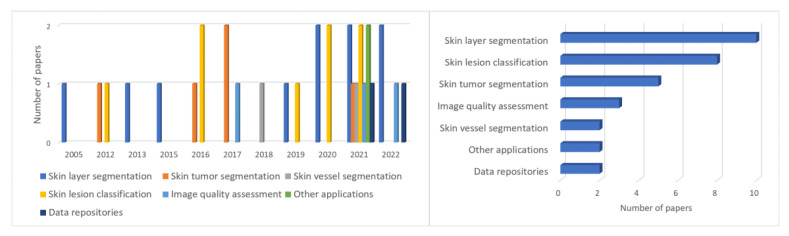
Timeline of the CAD algorithms developed for HFUS imaging analysis and their breakdown into categories based on application.

The CAD algorithms were divided into six categories based on the search results. These categories correspond to the subsections in the Methodology section of this survey. The growth in the number of papers in each category and the years of their publications are shown in Figure 1. Besides the six already mentioned groups of CAD problems, the timeline (Figure 1) includes HFUS data repositories, which are an integral part of the development of CAD systems.

All the solutions, followed by the numerical evaluations, were summarized in each category. The most promising and well-evaluated were marked and indicated as having potential for future development, along with their underlying limitations and further requirements.

To sum up, with this survey we aim to:

- show that methods using HFUS for skin analysis are now a developing area of image processing;
- quantitatively and qualitatively analyze all the developed methods;
- indicate the algorithms that are the most promising or have the potential for clinical usage in each category;
- discuss the limitations of the current approaches and future directions;
- highlight the specific contributions of the referred works;
- identify challenges in HFUS image processing.

The rest of the paper is structured as follows. Section 2 introduces the HFUS imaging technique and defines the range of sound wave frequencies considered “high”. A survey of the HFUS imaging devices applied for skin diagnosis that were described in the medical and technical works, with a particular emphasis on the latter, follows this introduction. Although still not commercially used, the potential of HFUS imaging systems for clinical diagnoses, as presented in the literature, is also discussed.

Since the review of the medical applications of HFUS was not the goal of our research, only the selected and most important works underlining the variety of HFUS usage in clinical practice are referred to in Section 3. They are summarized and compiled with the already developed CAD algorithms, which can support diagnosis in particular areas.

The review’s main section (Section 4) is divided into six subsections, representing the categories into which the CAD algorithms can be divided. Section 5 presents the HFUS image repositories and Section 6 is a critical discussion and an outlook for future research.

**Figure 2:**
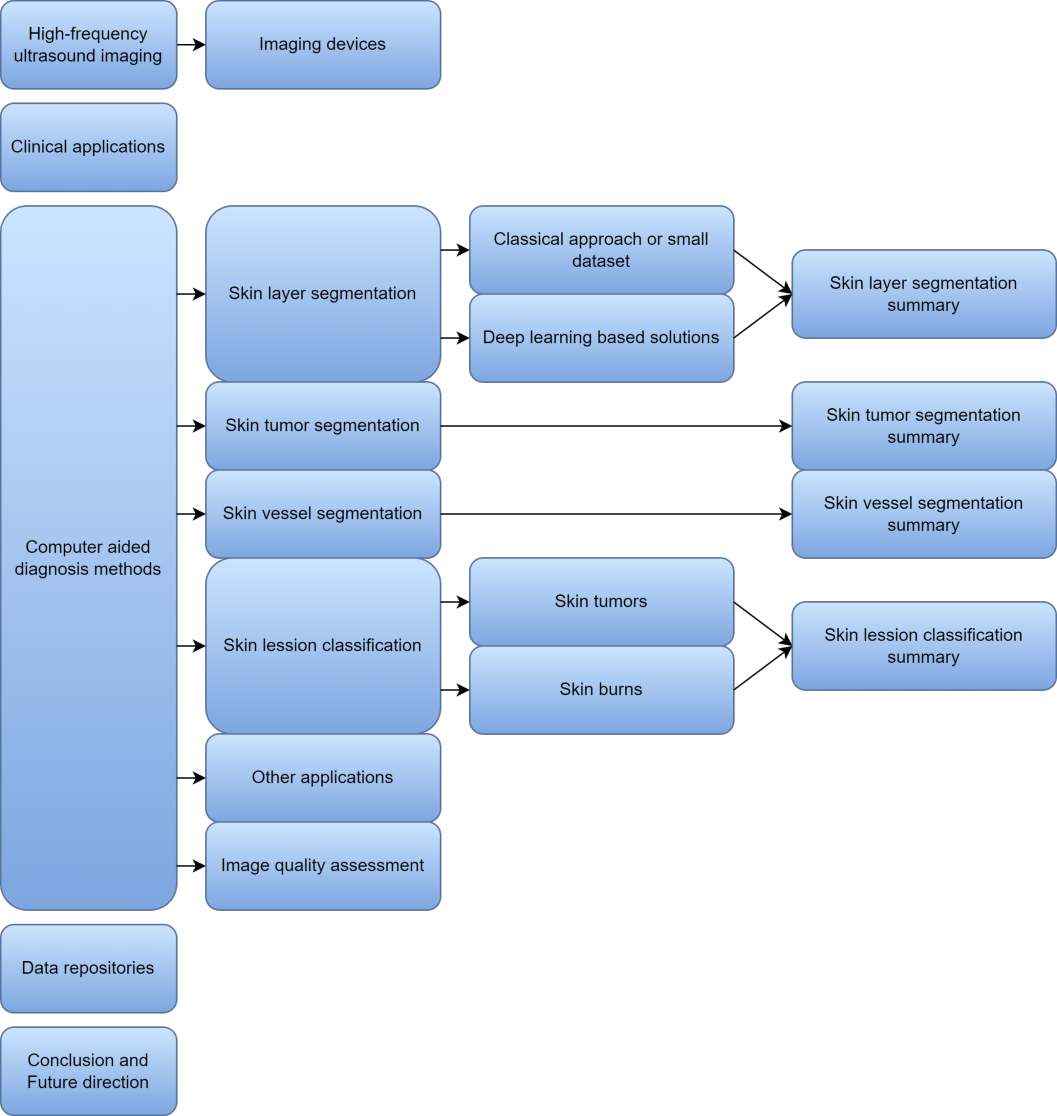
Review’s structure diagram.

The structure of our review is visualized in Figure 2.

## 2. High-Frequency Ultrasound Imaging

Ultrasound imaging, being cheap, noninvasive, and accessible, is one of the most important clinical diagnostic tools [5]. The name ultrasound (sonography) refers to the medical imaging technique of applying sound waves of 1–20 MHz frequency to view the inside of the body. It allows for differentiating tissues with only a 0.1% density difference [7]. The ultrasound penetration depth can reach 30 cm and its application areas continue to expand.

The development of ultrasonic imaging, together with growing interest in high resolution, opens up new diagnostic areas [8]. The higher probe frequencies (20–100 MHz) at the expense of a lower penetration depth (12–3 mm) provide relatively high resolutions, ranging from 80 to 16 μm, which are sufficient for skin structure visualization [7]. Almost all the HFUS scanners use one piezoelectric element (piezoelectric polymer PVDF or the copolymer P(VDF-TrFE)) [5,7]. The B-Scan is obtained as a stack of A-Scans reassembled in a two-dimensional image, where each part of the A-Scan corresponds to a tissue-specific point situated on the axis of the beam propagation [7].

The 20 MHz limit that separates classical scanners from high frequencies is contractual, and in different studies, it varies from 10 to 30 MHz. Shung [5] referred to 30 MHz; Bezugly [7], Grégoire et al. [9], and Czajkowska et al. [3] placed it at 20 MHz; Sciolla et al. [10] defined it as 15 MHz; and Bhatta et al. [6] reduced it to 10 MHz. The term ultra-high-frequency ultrasound (UHFUS) is also used for the range of frequencies between 30 and 100 MHz [11] and the term ultrasound biomicroscopy (UBM) is used for ultrasound probe frequencies above 50 MHz [6].

### Imaging Devices

The objective, quantitative method used to study normal skin and skin pathologies forms the basis of the development of several commercial systems. At least eight manufacturers produce HFUS machines for skin diagnosis and their image data are analyzed and described in the literature. They are partially listed in [12,13]. The first one is tpm, Taberna Pro Medicum (Lueneburg, Germany), which provides HFUS scanners with probe frequencies from 18 to 100 MHz. The offered penetration depth ranges are from 1.6 to 3.2 mm with a maximum axial resolution of 16 μm at 100 MHz for the DUB 100-12 bit device, and from 3.2 to 15 mm with a maximum axial resolution of 21 μm at 75 MHz for the DUB SkinScanner75. The analysis of the data acquired with the latter device (with 22 and 75 MHz probes) was described in [2,3,7,14,15,16,17]. The second company is Cortex Technology (Aalborg, Denmark), which provides the Dermascan C system used in [18,19]. There are two available transducers: 20 MHz, with a 60 by 150-micron resolution and in-depth penetration of 15 mm or a 60 by 260-micron resolution and a 23 mm penetration, as well as 50 MHz providing a 30 by 60-micron resolution and a 3 mm max penetration. The third manufacturer referred to in the literature [20,21] is Hitachi (Tokyo, Japan). The high-frequency machine HI VISION Preirus is equipped with a 5–18 MHz transducer (EUP-L75). However, due to the frequency <20 MHz, it is not widely described for skin diagnosis. The next one is the Dramiński, DermaMed (Gietrzwałd, Poland), which is equipped with a 48 MHz transducer. It is referred to by [22] as the gold standard. Another one is the Episcan I-200 that is provided by Longport Inc. (Chadds Fort, PA, USA) with 20–50 MHz transducers, which was applied in [23,24,25]. Its penetration depth ranges from 22.5 to 3.8 mm for the highest-frequency probe. The seventh system is the Atys Dermcup developed by Atys Medical (Soucieu en Jarrest, France) that was utilized in [10,26,27]. It is one of the oldest HFUS devices, with available probes of frequencies from 16 to 50 MHz, a penetration depth of 0–12 mm, and a maximum axial resolution of 30 μm. Additionally, FUJIFILM VisualSonics (Toronto, ON, Canada) offers the Vevo 3100 imaging system with a probe frequency of up to 70 MHz and a resolution of 30 μm. Its application in dermatology was described in [28] in the assessment of nodular skin melanoma Breslow thickness in adults. It was also successfully applied in cardiac diagnosis [29]. All the mentioned devices with their acquisition parameters are listed in Table 1 (the ‘-’ denotes that the company did not provide detailed information or the equipment is no longer being offered).

**Figure 3:**
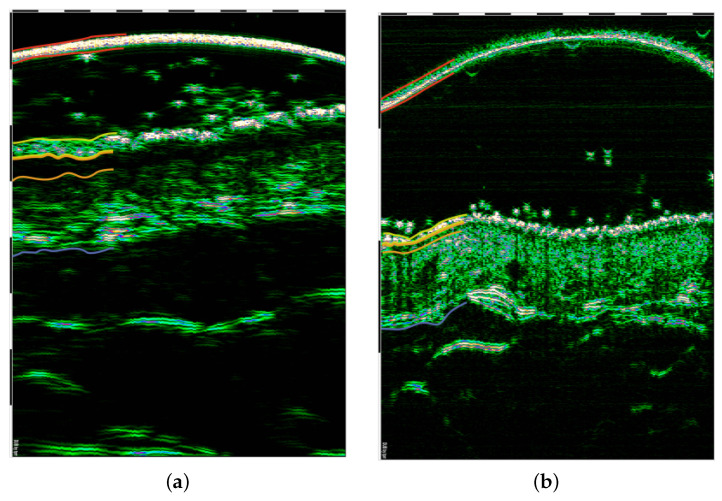
Skin images acquired using DUB SkinnScanner75, tpm (Lueneburg, Germany) [30] with different transducers: (**a**) 22 MHz, (**b**) 75 MHz.

A comparison of the skin images acquired using a DUB SkinnScanner75, tpm (Lueneburg, Germany) [30] with different transducers (22 MHz and 75 MHz) is shown in Figure 3. The colored lines partially delineate the most critical areas in the images. The red lines indicate the probe membrane, which is often visible in HFUS images; the yellow lines delineate the entry echo layer; the orange lines delineate the SLEB; and the blue lines are placed at the lower dermis edge.

In addition to those referred to above, there are different high-frequency ultrasound imaging systems described in the literature, which are still not commercially used [5,8,20,22,37,38]. One of the first was proposed by Berson et al. [37]. The wide-band 17 MHz center frequency transducer enables the acquisition of images with a resolution of about 0.08 mm in the axial direction and from 0.2 to 0.3 mm in the lateral direction. Next, Turnbull et al. [8] proposed the real-time ultrasound backscatter microscope operating in the 40–100 MHz range, providing an axial resolution of between 17 and 30 μm and a lateral resolution of between 33 and 94 μm. Devices in the frequency range of 20–80 MHz were investigated in [38]. High-frequency linear arrays and imaging systems in the 20–50 MHz range were developed in [5] to address the problem of mechanical motion and fixed focusing appearing in HFUS scanners. The newest work by Csány et al. [22] introduced a compact system using co-registered optical and ultrasound imaging to provide diagnostic information about the full skin depth. The maximum penetration depth of the scanner is 10 mm. In one of the previous works by Csány et al. [39], the authors also presented a portable ultrasonic device for skin imaging.

## 3. Clinical Applications

Due to the fact that HFUS imaging is increasingly used in medical diagnosis, there have appeared different studies describing its possible applications and limitations. According to the newest critical reviews on HFUS applications in clinical practice [4,13,40,41], it can be used in dermatology, allergology, aesthetic medicine, dermatological oncology, or rheumatology. In all these disciplines, the normal skin parameters, that is, layer thickness or intensities, can be used as a reference to compare with the abnormalities.

The primary dermatological use of HFUS is the assessment of skin cancers, including the pre-operative diagnosis and early detection of neoplasms [40]. The authors mentioned the characteristic features of basal cell carcinoma (BCC) manifested in HFUS. Similar to melanoma, the lesion’s shape correlates with its histologic subtype. The correlation of the Breslow thickness of melanomas in HFUS with histologic measurements is predicted to be over 92%. However, the clinical relevance of HFUS in examining cutaneous squamous cell carcinoma (SCC) is less well-defined and limited by the presence of artifacts. Vergilio et al. [4] mentioned the possible usage of HFUS in the diagnosis of cutaneous lymphomas, bullous pilomatricoma, extramammary Paget’s disease, Bowen’s disease, atretic cephalocele, and infantile hemangiomas. Dinnes et al. [42] assessed the diagnostic accuracy of HFUS when used to assist with the diagnosis of common pigmented skin lesions and their possible differentiation from melanoma. On the other hand, Polańska et al. [41] indicated the role of SLEB thickness measurements in diagnosing skin lymphomas since they may correspond to the severity of the disease. The probe frequencies in oncological application range from 15 to 50 MHz and a summary of the extracted tumor features is presented in [43].

One of the earliest applications of ultrasound is the assessment of inflammatory skin diseases [40,41]. In scleroderma, due to the increase in collagen deposition, it can differentiate between the inflammatory and sclerotic phases. Both in psoriasis and atopic dermatitis (AD), recent studies have indicated a reduction in the SLEB thickness as a parameter describing the treatment effects. In AD, the SLEB thickness correlates with the histological degree of epidermal hyperkeratosis, parakeratosis, spongiosis, and the intensity of inflammatory infiltrates, as well as the provider-assessed EASI (eczema area and severity index) scores [40,41]. As reported in [41], in patients with AD, the presence of the SLEB may be accompanied by the lower echogenicity of the other skin layers, and the thin SLEB area may also be present within healthy skin around the affected region. In psoriasis, apart from the presence of the SLEB, a thickened and streaky entry echo layer perpendicular to the entry echo shadows may be detected [41]. HFUS is also used in the diagnosis of hidradenitis suppurativa, enabling the detection of many lesions missed in palpation. The newest study by [40] reported a strong correlation between the mean brightness of HFUS and the lesion CSAMI (cutaneous sarcoidosis activity and morphology instrument) score in sarcoidosis.

As reported in [40,41], skin thickness is inversely proportional to age, and the skin thickening in children and young adults may be an important indicator of the presence of inflammation or other pathological conditions (e.g., morphea or the sclerodermic variant of chronic graft). Additionally, in allergology, during the evaluation of a patch test, the increase in skin thickness correlates with the intensity of the allergic reaction [41].

In cosmetology and aesthetic medicine, HFUS is used for skin-aging assessments, where the most sought-after features are the arrangement of collagen and elastin bundles, water loss, skin thickness, and presence/parameters of SLEB, which tend to change with age and are sensitive to botulinum toxin injections or topical vitamin C therapy. Vergilio et al. [13] presented a comparison of photo-exposed and photo-protected regions of mature skin and the different aging of the skin in these regions, a comparison of skin aging with respect to sex, and a comparison of oily and normal/dry-skin aging. The newest work by [4] complemented the HFUS application of cellulite, dermal fillers, tattoo reactions, hypertrophic scars, and acne vulgaris. The works cover a wide range of applied frequencies from 15 to 100 MHz.

According to [40], HFUS allows for differentiation between abnormalities in glabrous skin in conditions such as Pachyonychia Congenita and other palmoplantar keratodermas. It can be used to estimate the phase of hair follicle growth, identify the inflammation of hair follicles, assess hair density, and characterize scalp cysts. It is also applicable in the diagnosis of nail diseases.

To summarize this section, in Table 2, we presented the medical problems that have appeared in the literature, which can be overcome by the already developed CAD methods described in detail in the following sections. According to this summary, the CAD algorithms can assist in the diagnosis of skin tumors by measuring the depth of their penetration, extracting specific features from the segmented areas, or even differentiating between tumor types. They support the dermatological diagnosis of inflammatory skin diseases by classifying them or measuring skin layer thickness in the affected regions and their surroundings. The same skin layer segmentation algorithms can be used in aesthetic medicine, cosmetology, or allergology. In addition to the applications mentioned above, a HFUS data analysis concerning its accuracy may help in a dedicated training program.

As reported in [40,41], the use of HFUS in the final diagnosis of tumor type and the differentiation between benign and malignant tumors on an ultrasound pattern is not completely possible. However, some authors have postulated these options, and HFUS image analysis may show features that facilitate lesion recognition. Therefore, some additional research, more clinical data, and strong cooperation between medical and technical teams are strongly recommended in this area.

## 4. Computer-Aided Diagnosis Methods

Currently, the development of computer-aided diagnosis (CAD) methods involves already-developed imaging systems. Therefore, a fast and robust diagnosis based on medical images is always connected with this type of system, and due to their rapid development, CAD systems have become a part of routine clinical work [56].

CAD systems are designed to assist doctors in interpreting medical images. They process digital images such as chest X-rays, computed tomography (CT), mammography, ultrasound, colonoscopy, etc., to highlight conspicuous sections, indicate pathology, and segment lesions and classify or measure them, providing decision support. The algorithms for mass detection in mammography [57], lung cancer screening with computed tomography [58], and the detection of colonic polyps in CT [59] colonography are the most widely explored CAD algorithm applications. The latest target is COVID-19 diagnosis support [60] or digital pathology [61].

Different CAD algorithms dedicated to HFUS image analysis have also appeared in recent decades. They cover the essential aspects of skin diagnosis such as skin layer and lesion segmentation or HFUS skin image classification. All of them are divided into categories and are described in the following sections. Additionally, Table 2 presents their connections with clinical applications, where the last column indicates the paper describing the proper algorithm.

Different quality measures were applied depending on the processing task (segmentation or classification), which covered various aspects of the analysis. Moreover, the authors used other measures to validate their work so finding a standard solution in these studies was problematic. Due to this, for the following sections, we decided to consider the most frequently utilized quality measures to enable easy comparison of the reported results or the most informative in the case of the absence of the first choice measure.

For image segmentation, there were three measures we utilized. The first one was the Sørensen–Dice coefficient (Dice index) [62], which measures the similarities between two sets of data.

The second one was the symmetric mean absolute distance (MAD), calculated as the mean of the shortest distance between points at the boundary of the segmented object to the reference mask and vice versa [63]. The last one was the directed Hausdorff distance (HD), which describes the maximum distance from a point in the first set to the nearest point in the other.

In addition to these three measures, both Lagarde et al. [27] and Gao et al. [64] used the correlation coefficient $R^{2}$ to compare manual measurements of dermis thickness with automated or semi-automated techniques. Since the authors did not provide any of the aforementioned segmentation quality measures, the correlation coefficients were also considered in our analysis.

For image classification, we selected the four measures most frequently referred to in the cited works: sensitivity (TPR), specificity (TNR), accuracy (ACC), and area under the ROC curve.

Table 3 describes colour nomenclature, being used for color-coded Table 4, Table 5, Table 6, Table 7, Table 8, Table 9, Table 10 and Table 11, which are included in the following sections and summarize the CAD methods in the HFUS of skin tissues. The colored rows indicate the most interesting solutions and the tones indicate their importance. This mainly reflects the highest accuracy obtained in the summarized category. The number of analyzed examples was also considered in the case of similar scores or a significant difference in the size of the studied populations.

**Figure 4:**
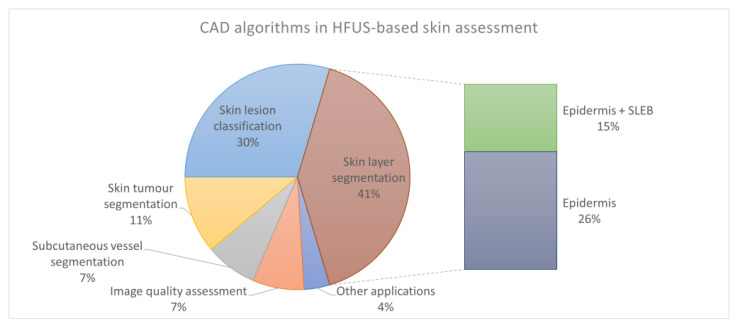
CAD applications in HFUS image analysis.

Based on Table 2 and Table 4, Table 5, Table 6, Table 7, Table 8, Table 9 and Table 10, which are presented in the next sections, as well as Figure 4 (summary of individual applications for CAD of skin), we concluded that the most widely explored area was skin layer segmentation [3,10,14,15,16,17,27,48,64,76]. However, the majority of the works limited the analysis to the epidermis region, which is often crucial for further automated processing steps but is not sufficient for a complete diagnosis.

**Table 7:** Skin vessel segmentation summary.

Authors of the Algorithm/Implementation	Algorithm	HFUS Device	Nb. of Cases	Results
Mathai et al. [79]	distance-regularized level set	Vevo 2100, FUJIFILM Visual Sonics [32] (Toronto, ON, Canada) [32]/Diasus, Dynamic Imaging (UK) 10–22 MHz	35 sequences (3500 images)/5 sequences (1250 images)	mean(D) = 0.911±0.019
	**Contribution:** new distance-regularized method for subcutaneous vessel segmentation; extensive database; the highest segmentation accuracy; manual seed-point selection; lack of repeatability analysis; **Disadvantages:** single imaging device			
Pyciński et al. [80]	geometric active contour	DUB SkinnScanner75, tpm (Lueneburg, Germany) [30], 22 MHz [30]/iU22, Philips (Amsterdam, Holandia), L12-5 12 MHz	54 HFUS images/12 US images	HFUS: mean(D) = 0.9, min(D) = 0.77, max(D) = 0.95, US: mean(D) = 0.87, min(D) = 0.71, max(D) = 0.94
	**Contribution:** new active contour model with edge constraints for subcutaneous vessel segmentation; high segmentation accuracy; universal analysis method (US and HFUS images); manual seed-point selection; lack of repeatability analysis; **Disadvantages:** limited database			

**Table 8:** Skin lesion classification summary (part 1).

Authors of the Algorithm/Implementation	Algorithm	HFUS Device	Nb. of Cases/Lesion Type	Results
Kia et al. [81]	multilayer perceptron	DUB SkinnScanner75, tpm (Lueneburg, Germany) [30]-the authors did not provide this information	120 images/healthy, benign, BCC, Melanoma	TPR = 0.98, TNR = 0.05
	**Contribution:** new application area for multilayer perceptron; **Disadvantages:** single imaging device; missing acquisition protocol; low specificity			
Csabai et al. [82]	AdaBoost/SVM	HI VISION Preirus, Hitachi (Tokyo, Japan), 5–18 MHz (EUP-L75) [36]	248 images: 73 melanomas, 130 BCCs, 45 benign nevi	BCC vs. nevus (SVM): AUC = 0.90, TNR = 0.45, Nevus vs. others (SVM): AUC = 0.86, TNR = 0.19, Melanoma vs. nevus (AdaBoost): AUC = 0.88, TNR = 0.26
	**Contribution:** new application area for AdaBoost and SVM; extended dataset compared to previous work; **Disadvantages:** single imaging device; low specificity			
Andrekute et al. [83]	SVM	DUB SkinnScanner75, tpm (Lueneburg, Germany) [30], 22 MHz [30]	160 datasets: 80 melanomas, 80 benign melanocytic nevi	TPR = 0.824, TNR = 0.858
	**Contribution:** new feature set definition for SVM analysis; **Disadvantages:** single imaging device; limited dataset; low accuracy			
Kia et al. [45]	DCT + neural network	50 MHz, the authors did not provide additional information	400 images/healthy, benign, BCCs, melanomas	TPR(2 groups) = 0.938, TNR(2 groups) = 0.973, AUC(2 groups) = 0.859
	**Contribution:** new application of DCT and neural network for skin lesion classification; **Disadvantages:** single imaging device; missing acquisition protocol; two-group analysis			
Kia et al. [81]/Kia et al. [45]	multilayer perceptron	50 MHz, the authors did not provide additional information	400 images/healthy, benign, BCCs, melanomas	ACC (4 groups) = 0.917
	**Contribution:** application of previous approach to new classification problem; multiclass analysis; quite high accuracy for 4 groups; **Disadvantages:** single imaging device; missing acquisition protocol			
Tiwari et al. [46]	SVM	DUB SkinnScanner75, tpm (Lueneburg, Germany) [30], 22 MHz, optical dermatoscop and spectrophotometer SimSys, MedX Health (Malton, ON, Canada) [84]	91 images/41 malignant melanomas, 50 melanocytic nevi	ACC = 0.989, AUC = 0.999, TPR = 0.975, TNR = 1.000
	**Contribution:** new feature set for SVM classifier; the highest accuracy for 2 groups; **Disadvantages:** single imaging device; quite small dataset			
Marosán-Vilimszky et al. [21]	SVM	HI VISION Preirus, Hitachi (Tokyo, Japan), 5–18 MHz (EUP-L75) [36]	310 images/70 melanomas, 130 BCCs, 110 benign nevi	BCC vs. nevus: AUC = 0.921, AUC(LAR) = 0.957 Nevus vs. others: AUC = 0.914, AUC(LAR) = 0.953 Melanoma vs. nevus: AUC= 0.896, AUC(LAR) = 0.933
	**Contribution:** new feature set for SVM classifier; representative dataset; high accuracy for 2 groups; **Disadvantages:** single imaging device			

**Table 9:** Skin lesion classification summary (part 2).

Authors of the Algorithm/Implementation	Algorithm	HFUS Device	Nb. of Cases/Lesion Type	Results
Huang et al. [85]/Czajkowska et al. [74]	DenseNet-201 TL Grad-CAM	DUB SkinnScanner75, tpm (Lueneburg, Germany) [30], 75 MHz [30]	631 images: 200 non-melanocytic skin tumors, 303 AD, 77 psoriasis, 51 healthy skin	ACC= 0.975
	**Contribution:** first application of DenseNet to HFUS image classification; DNN model evaluation/interpretation; large dataset; different diseases; **Disadvantages:** only 4 different groups considered; single imaging device			
Lee et al. [86]	SVM	Ultrasonix (USA) L40-8/12, 10 MHz	143 images: 37 burn group (i), 33 burn group (ii), 39 burn group (iii), 34 burn group (iv)	ACC = 0.93
	**Contribution:** first application of SVM to burns HFUS image classification; high classification accuracy; **Disadvantages:** phantom study; single imaging device			

**Table 10:** IQA of HFUS images.

Algorithm	HFUS Device/Data	Number of Groups	ACC	F1-Score
VGG16 [87]	DUB SkinnScanner75, tpm (Lueneburg, Germany) [30], 24 MHz /17,425 images of facial skin [88]	2	0.8907–0.8999	0.8716–0.8993
DenseNet-201 [85]	DUB SkinnScanner75, tpm (Lueneburg, Germany) [30], 24 MHz /17,425 images of facial skin [88]	2	0.8682–0.8802	0.8480–0.8800
VGG16 + MIS [89]	DUB SkinnScanner75, tpm (Lueneburg, Germany) [30], 24 MHz /17,425 images of facial skin [88]	2	0.9170	0.9076
VGG16 [87,89]	DUB SkinnScanner75, tpm (Lueneburg, Germany) [30], 24 MHz /17,425 images of facial skin [88]	4	0.8284	0.8401

**Table 11:** HFUS image datasets available in the public domain.

Name	Repository	Content/HFUS Device	Number of Cases	Number of Patients
Data for Deep Learning Approach to Skin Layer Segmentation in Inflammatory Dermatoses [67]	Mendeley Data [90]	inflammatory skin diseases, image segmentation: SLEB, epidermis; single expert delineations/ DUB SkinnScanner75, tpm (Lueneburg, Germany) [30], 75 MHz	380: 303 AD, 77 Psoriasis	380
High-Frequency Dataset of Facial Skin [88]	Mendeley Data [90]	facial images, classification, two experts annotations; IQA/ DUB SkinnScanner75, tpm (Lueneburg, Germany) [30], 75 MHz	17,425	44

The second most widely explored area was skin tumor segmentation [20,26,63,75,76,78]. In support of skin tumor diagnosis, there also appeared works targeting HFUS image classification [21,45,46,81,82,83], with a particular emphasis on melanocytic lesion classification. However, other classification problems were also found in the literature [74,86].

Some works addressed the problem of subcutaneous blood vessel segmentation [79,80] or HFUS image quality assessment [55,89]. A novel trend in CAD algorithms is sharing the datasets or source code [67,88,91].

### 4.1. Skin Layer Segmentation

Due to the fact that the segmentation algorithms are among the most explored in medical applications and the universality of their solutions enables their application in various areas, the HFUS image segmentation methods, among other CAD solutions, were historically first addressed in the literature in [27,64]. At the same, they are the most widely described and evaluated algorithms [10,14,26,27,63,64,92].

According to skin morphology, skin layer segmentation is the most functional area of interest and the clinical applications of HFUS prove this need [13,40]. In inflammatory skin diseases, the presence of SLEB and its thickness measurements is an indicator of treatment effectiveness [19]. The acoustic density of the segmented layers has been considered in cosmetology and aesthetic medicine [51], and the characterization of the dermis due to age was addressed in [13].

#### 4.1.1. Classical Approach or Small Datasets

The first skin segmentation algorithm for HFUS images was developed by Lagarde et al., and was described in [27]. This study aimed to segment the dermis layer for its thickness estimation automatically. As the authors claimed, in this application, the analysis helped to evaluate dermo-cosmetics for assessing both the morphological changes and mechanical properties of this layer. An important novelty of this work was the standardization of the manual dermal thickness measurement procedure on B-scan ultrasound images. The layer boundaries were detected using a modified active contour algorithm [93]. To evaluate the developed tool, the authors used 612 HFUS images acquired using an Atys Dermcup, Atys Medical (Soucieu en Jarrest, France) [35], at 20 MHz. The image processing algorithm was compared with both the manual and semi-manual measurements. In addition, the intra-operator variability was measured to check whether the computerized results placed in the range of the expert mistakes.

The second work [64] targeted dermis segmentation to assess skin toxicity measurements. The developed algorithm utilized the benefits of curve evolution under the Riemannian metric [94]. The system was tested on a breast cancer radiotherapy ultrasound imaging database consisting of 730 HFUS images of 23 patients.

The first application in this area by Sciolla et al. [10] targeted both skin layer and skin lesion segmentation. This preliminary study was extended in [63]. It examined the problem of dermis segmentation in 50 MHz images. The practical goal of the analysis was to study skin photoaging. The joint epidermis and dermis segmentation method applied a non-parametric active contour method level set, which combined a texture criterion and an epidermis indicator map with the geometric constraint from the layered morphology. The SLEB was also characterized in this work based on the segmented dermis area cut into slices and the statistical analysis of the ultrasound envelope signal. However, its accurate segmentation was not evaluated in this study. The analyzed dataset consisted of HFUS images of 76 healthy women acquired on the external face of the left forearm. The thorough analysis of the results included a comparison with other promising segmentation methods described in the literature [64,65] utilizing the active contour models. However, the numerical analysis of the segmentation results was limited to 20 images, which two experts manually delineated in the dermis part (joint epidermis and dermis). The obtained joint epidermis and dermis segmentation masks were compared with manual delineation using MAD and the Dice index. The numerical results obtained for the reference methods [64,65] are included in Table 4. The rest of the input data were analyzed for the visual score of skin aging (SCINEXA score [95]).

The extraction, modeling, and quantification of skin layers was the target of the work [92] by Bryjova et al. The segmentation method utilized a mathematical model of skin morphology based on the skin layer skeleton, and the study aimed to assess burn treatment. To verify the segmentation method, the authors used a Mindray M7, Mindray (Shenzhen, China) machine with an 11 MHz transducer. Since this machine is not typically dedicated to skin diagnosis, it is not mentioned in the device list in Section 2. A quantitative evaluation did not follow the somewhat preliminary work.

The preparatory work by Czajkowska et al. [14] in skin analysis targeted wound treatment assessment. The developed methodology consisted of fuzzy c-means (FCM) clustering followed by signal analysis, where the one-dimensional data were the coordinates of the pre-segmented epidermis layer. The algorithm was applied to 13 HFUS images acquired with a 22 MHz transducer. The results were compared with manual expert delineations (provided by two independent experts) using HD and the Dice index (see Table 4).

The promising preliminary results encouraged the authors to develop the skin layer segmentation methods, and the next paper [15] we discuss targeted both the epidermis and SLEB layer analysis. It was the first work in CAD methods of inflammatory skin diseases based on HFUS images. The developed technique extended the level set [96], introducing a gradient field based on FCM clustering. The 45 HFUS images employed in this study were acquired using a DUB SkinnScanner75, tpm (Lueneburg, Germany) [30] (75 MHz) and were manually delineated by two experts. The measured thickness of the segmented bands and the spatial overlaps of two segmented regions were compared with the measurements and masks resulting from the expert delineations. Statistical analysis was introduced to evaluate the hypothesis that the obtained results were comparable to those provided by the experts. The authors concluded that the automated segmentation results were similar to those generated by the experts. According to the summary in Table 4, these results outperform those generated by the previously discussed methods. However, due to the different transducer frequencies resulting in different image resolutions and the various datasets used in the analysis, a direct comparison of the results should be avoided. Moreover, the same methodology was verified on the comprehensive dataset (380 images) in [3], resulting in much lower accuracy (median(D) = 0.544).

The fast development of knowledge-based methods introduced by deep neural networks (DNN) has enabled their implementation in HFUS image segmentation. The preliminary work in this area [16] utilized the U-Net model developed by Ronneberger et al. [66] to segment the epidermis and SLEB in 75 MHz ultrasound images. It was an extension of the previous work [15] by these authors in the area of CAD of inflammatory skin diseases. To increase the number of training samples, the authors divided each image into several subimages, treating each of them as a separate input. The authors ensured that a single patient’s subimages were not separated into training and testing sets. The obtained results are comparable to those previously described in the literature [10,15,63]. Despite the DNN solutions being addressed in the next section, due to the small dataset considered in this work, we decided to discuss the work [16] here.

Skin layer segmentation was also an intermediate step for the skin lesion detection described in [20]. Similar to [97], the segmentation step was carried out in a few stages. It utilized median filtering, Otsu thresholding, heat map construction, and the parametrized active contour model [94]. Since the authors focused on skin tumor segmentation, their work did not validate the skin layer segmentation aspect.

All the already mentioned skin layer segmentation methods are summarized in Table 4. They all provide acceptable accuracy; however, the experiments were limited to small datasets, and the different acquisition parameters used do not allow for their direct comparison.

#### 4.1.2. Deep Learning-Based Solutions

The main problem with implementing DNN for image segmentation is the lack of training data. In addition, access to more extensive datasets containing expert delineations [88] opens up a new area in image analysis, understanding, and segmentation. The continuation of the work by [16] in the field of HFUS image segmentation in the CAD of inflammatory skin diseases was described in [3]. The paper presented a novel framework for both epidermis and SLEB analyses. The developed SegUnet model was extended by a pre-processing step utilizing the FCM results and L* a* b* color space [98] as the network inputs. The SegUnet architecture used the encoder–decoder model proposed by Ronneberger et al. [66] and a batch normalization layer, which was adopted from SegNet [68]. As the authors claimed [3], it adjusted and scaled the activation, made the segmentation results more stable, and increased the neural network performance. The analyzed dataset [67] consisted of 380 HFUS images of 380 different patients with inflammatory skin diseases: AD (303) and psoriasis (77). The data were acquired using a DUB SkinnScanner75, tpm (Lueneburg, Germany) [30] at 75 MHz. The data were annotated and manually delineated by a single expert; however, the outcomes were verified by another expert. In their experiments, the authors compared the designed model with others described in the literature as accurate in layered object segmentation [69]. The considered Ce-Net model [69] was successfully applied for retina layer segmentation in OCT images.

It is worth mentioning here that the similarity of OCT images of the skin or retina to skin HFUS data suggests that the methods used for their analysis [99,100,101,102] could be adapted to the segmentation of skin HFUS images. Similar, the algorithms targeting skin segmentation in hist-pat images [103,104,105] can be an inspiration for HFUS dedicated frameworks.

The experiments described in the work by [3] included numerical analysis of the different DNN models depicted in the literature. Those with the potential for skin layer segmentation are listed in Table 5, where the usage of DNN as a tool for skin layer segmentation is summarized. All the models [3] were trained from scratch, and two variants of input data were evaluated. The first analyzed the original RGB images, whereas the second modified the input space, as described above. This modification improved the segmentation results for most investigated models (CE-Net, U-Net, and SegUnet). To increase the number of training data, the input images were augmented by introducing random geometric transformations (reflection in the horizontal direction, ±10-pixel translation in both directions, $\pm 10^{\circ }$ rotation). However, the authors did not provide a quantitative analysis of the influence of the augmentation step. Each training process consisted of 200 epochs, and a discussion concerning interrupting the training process using a validation set was also skipped. They trained the network by optimizing the cross-entropy (CE) loss through the stochastic gradient descent with a momentum (SGDM) optimizer and training data were shuffled before each training epoch. The batch size was 8, and the initial learning rate equaled 0.001 for all the analyzed DNN models.

The application of the newest U-shaped models, basic U-Net by Ronnenberger et al. [66], DC-UNet [71], and CFPNet-M [72], to epidermis and SLEB segmentation can be found in [48]. The authors analyzed the influence of the size of the images used for network training, augmentation technique, optimization method, region of interest (ROI) selection, and binarization threshold on the final segmentation accuracy. During the experiments, they used external *k*-fold cross-validation and analyzed the impact of the *k* value on the obtained results. Based on the outcome of the investigations, we can conclude that surprisingly, the ROI reduction did not continuously improve the segmentation results. Moreover, the authors recommended the Adam optimizer, which outperformed the SGDM in the final segmentation results. An interesting observation is connected with the augmentation step, according to which the limited rotation works better for this type of image (layered). The best results obtained by each of the models investigated in this work [48] are included in Table 5.

The next work by Czajkowska et al. [74] described epidermis segmentation as a part of the HFUS skin image classification framework. The analyzed image set was extended (compared to [3,48]) by 200 images of non-melanocytic skin tumors. However, because the SLEB layer was not present in patients with skin tumors, it was omitted in this analysis. The epidermis segmentation step created a skin layer map defining the region of interest, which was then correlated with the DNN network (applied for classification) response. Among many other solutions, the authors selected the DeepLab v3+ model [73] with a pre-trained Xception [106] backbone as the most accurate solution.

The last work in this area [17] also limited the segmentation step to the epidermis region. The authors [17] focused on an accurate, robust, and repeatable segmentation including details at the object borders. For this, they combined deep models with the fuzzy connectedness (FC) analysis developed by Udupa et al. [107] for fine segmentation. Similar to [48], the authors considered the ROI selection step and the experimental results favored it in this case. The pre-processing step targeting the ROI selection utilized a DeepLab v3+ [73] network built on a pre-trained backbone for feature extraction. From the three analyzed backbone models, the authors selected a 50-layer residual backbone (ResNet-50) as the most efficient in a series of experiments. The same architecture was chosen for the final epidermis segmentation step. To justify the FC parameters, they utilized the interpolated Dice index heatmap. In the experiments, they considered different state-of-the-art techniques [3] trained over the benchmark database [88]. Additionally, they applied FCN-AlexNet and FCN-8 s, pre-trained on CamVid and ImageNet datasets with different optimizers (SGDM and Adam) and loss functions (CE and Dice loss). Table 5 presents the most promising results out of all the experiments.

#### 4.1.3. Skin Layer Segmentation Summary

Skin layer segmentation was the most widely explored aspect of the CAD systems described in this paper. The essential findings in this area can be summarized as follows:

- Two main branches were visible in this field: deformable models and deep neural networks.
- Due to the different application areas, ultrasound machines used during image acquisition, and various frequencies of the transducers, a direct comparison of the algorithms and quantitative analysis of their results was hard or even impossible.
- A few classical methods deserve special attention: [10,15,17,48]. The first two [10,15] applied level-set techniques, which resulted in a Dice index equal to 0.94 and 0.878, respectively, which is a high result for medical applications. The Dice index equal to 0.878 described in [15] places this work far behind [10]. However, the obtained mean absolute distance value (8.5 μm) was, in this case, much lower than that calculated by Sciolla et al. (45 μm), and this parameter seems to be more relevant for clinical diagnoses.
- Among all the CNN-based solutions, that in [48] is particularly noteworthy, as the authors applied the common U-shape models for both the epidermis and SLEB segmentation. The obtained accuracy (median(D) ≥ 0.93) for the DC-UNet model developed by Lou et al. [71] is at the level of clinical acceptance.
- Most of the listed solutions were dedicated to specific medical problems and it was hard to check their generalization ability.
- Since the authors did not present the whole datasets or representative samples visualizing the differences in the analyzed images, it was hard to assess the adaptability of the described methods to other types of data. For example, the level-set method described in [15] when applied to a bigger dataset (380 images [67]) in [3] resulted in a median Dice index equal to 0.727 for the epidermis and 0.54 for the SLEB. For the comprehensive dataset analyzed in [17], which included different diseases, the CNN-based segmentation results were lower than for the inflammatory one.
- The algorithms were trained and tested using the same scanners, and analyses of the various scanners are required for clinical usage.
- CNN-based analysis (especially using pre-trained models) requires image size reduction and the resulting mask needs to be enlarged to the initial size. This post-processing step can strongly influence the final accuracy. In [48], this step was skipped. The reader might assume that the resulting masks were compared with expert delineations in the initial space; however, he/she cannot be sure.
- Most reviewed solutions were fully automated and repeatable.
- The CNN model trained for skin layer segmentation, as well as the analyzed dataset described in [3], are available via Mendeley Data and enable the repeating of the experiments.

### 4.2. Skin Tumor Segmentation

Apart from skin layer analysis, HFUS imaging has opened up new possibilities in diagnosing skin tumors. The information on the tumor depth, vascularization, and morphology of adjacent tissues is the perfect complement to classical dermatoscopy. Fast, accurate, repeatable, and robust segmentation of skin tumors seems to be the natural direction of the developed CAD algorithms. Unfortunately, only a few papers have addressed this problem [10,20,26,76].

The segmentation of skin lesions in 2D and 3D ultrasound images was first described in [75]. The paper addressed the problem of jointly estimating the statistical distribution and segmenting lesions in skin ultrasound images. To model the tissues, the authors applied a heavy-tailed Rayleigh mixture based on the single-tissue model provided by Pereyra et al. [108] for modeling the ultrasound echoes in skin tissues. The novel approach incorporated the Markov random field (MRF) to model the spatial correlation of the considered biological regions and the Bayesian model for segmentation. The twofold validation method introduced synthetic and clinical data and the numerical results are available in Table 6.

The second work [10] by Sciolla et al., was a preliminary study and targeted non-homogeneous skin tissue segmentation in HFUS 3D images of the skin. The adaptive log-likelihood (AdLL) 3D level-set segmentation algorithm maximized the log-likelihood of a segmented contour. The performance of the developed methodology was evaluated for eight lesions diagnosed using an Atys Dermcup, Atys Medical (Soucieu en Jarrest, France) [35] 50 MHz system. Since the Dermcup device is equipped with a single transducer element, the 3D images are made of scans acquired along two orthogonal axes with the controlled transducer position. To provide a quantitative analysis of the developed method, the authors additionally produced synthetic 3D skin images. The segmentation results were compared with the expert delineation provided for single orientation and interpolated between slices. A summary of the obtained results can be found in Table 6.

An extension of this work was presented in [26]. It considered the two most common types of skin cancer: melanoma and basal cell carcinoma. The 3D HFUS image segmentation algorithm utilized a hybrid geodesic active contour with both area and boundary constraints. The developed probabilistic boundary expansion term was based on feature asymmetry (FA) according to [94] and the boundary term was curvature dependent. The parameters of each method were optimized to obtain the best average Dice index over the analyzed dataset. Similar to [76], the weak point of the processing framework was the manual seed-point selection (or ROI specification [76]) and the lack of discussion of its influence on the final segmentation results. The methodology was evaluated based on the 50 MHz 3D clinical images acquired using the Atys, Atys Medical (Soucieu en Jarrest, France) imaging system (three BCC and nine melanomas). As the authors mentioned, the set of 3D data consisted of 1800 2D images. The performance of the segmentation framework was compared with the manual expert delineations (prepared similarly to [76]) and the results are included in Table 6.

The problem of seed-point selection was solved in [20]. Based on the segmentation results mentioned in Section 4.1.1, the fully-automated framework utilized FCM clustering and a two-component heat map to find the lesions. The final segmentation step was based on the active contour model designed by Chan and Vese [65]. The evaluation step considered two data sources: a commercial high-frequency ultrasound imager HI VISION Preirus, Hitachi (Tokyo, Japan [36]), with 5–18 MHz EUP-L75 transducer and a custom system based on a manually scanning single-element transducer (V317, Olympus, (Tokyo, Japan)) [39,109], which included 60 images. The dataset was divided into 40 images used for parameter adjustment and 20 for validation. The evaluation step was limited to comparing the segmentation results obtained by the manual and automated seeding selection without discussing the primary segmentation technique.

The newest framework developed by Marosán-Vilimszky et al. [21] utilized the benefits of the seeding selection step and segmentation algorithm described in [20] for the skin lesion classification problem and compared it with the semi-automated approach.

A dataset concerning skin tumors was also analyzed in [17] but the segmentation step was limited to the epidermis layer and was discussed in Section 4.1.2.

An interesting extension of the works mentioned above was [110]. The authors presented an automated melanocytic skin tumor thickness estimation method based on a time-frequency analysis of US radio frequency signals. The image data were acquired using a DUB SkinnScanner75, tpm (Lueneburg, Germany) [30] (22 MHz probe). The sensitivity and specificity of the automated measurements compared with the histologic results were 96.55% and 78.26%, respectively, and compared with the manually measured US thickness were 75.86% and 73.91%.

#### Skin Tumor Segmentation Summary

The main findings in this area can be summarized as follows:

- Due to the small datasets and time-consuming manual expert analysis, the developed methodologies were limited to classical approaches.
- Similar to skin layers, the most promising were the active contour models, from which the highest accuracy resulted in the hybrid geodesic active contour with a probabilistic boundary expansion term described in [26].
- The small testing set and image acquisition limited to a single scanner, together with low accuracy, do not allow for its clinical usage at this stage.
- Most of the developed approaches required manual starting point selection and their repeatability in connection with this choice was not discussed in the papers.

### 4.3. Skin Vessel Segmentation

In addition to neoplastic lesions, the analysis of which is usually the most widely described in the literature or works addressing the challenges in the diagnosis of inflammatory skin diseases, incidences of which increase every year, the latest CAD systems also include algorithms for the segmentation of blood vessels [79,80] in HFUS images.

The first work by Mathai et al. [79] targeted the segmentation and tracking of small and medium hand vessels based on UHFUS images (above 50 MHz). The developed GPU-based approach enabled fast submillimeter 2D vessel contour localization. It was a combination of a local phase analysis providing robust edge detection and a distance regularized level set for contour segmentation followed by an extended Kalman filter (EKF) for the tracking step. The authors utilized a bilateral filter to smooth the slight amplitude noise, preserving the vessel boundaries. Next, the clustering approach [111] was used to find the seeds to track over sequential B-scans. The validation step included 35 UHFUS sequences (100 images each) acquired using a Vevo 2100, FUJIFILM Visual Sonics [32] (Toronto, ON, Canada) machine equipped with a 50 MHz transducer and 5 HFUS sequences (10–22 MHz, 250 images each) from Diasus, Dynamic Imaging (UK). Using common metrics, the segmentation results were compared with the annotations provided by two graders (the authors did not mention whether the graders were experts or non-experts). The vessel segmented in all B-scans of a sequence, which indicated that the tracking was successful.

As the authors reported, the obtained mean Dice scores for the UHFUS images were mean(D) = 0.917±0.019 against the first grader and mean(D) = 0.905±0.018 against the second grader, which were better than the inter-grader scores. For the HFUS data, the obtained scores were comparable with the inter-grader analysis.

The second work [80], a preliminary study by Pyciński et al., addressed the problem of vessel segmentation in both HFUS and US images and the overall goal of the study was a fusion of both these modalities. The segmentation step utilized color space transformation, anisotropic filtering, and WFCM (weighted FCM) clustering, followed by the geometric active contour [65], providing the final segmentation results. The basic registration covered only in-plane translation as the images were collected from the same anatomical plane. This was based on the correspondence of the centroids of the previously segmented regions. During the experiment, 54 HFUS images acquired using a DUB SkinnScanner75, tpm (Lueneburg, Germany) [30] with a 22 MHz transducer and 12 images using an iU22, Philips (Amsterdam, Holandia) with a linear transducer L12-5 of frequency 12 MHz were considered. The segmentation accuracy was estimated by comparing the obtained results with the manual expert delineations. The mean Dice index was equal to 0.9 for the HFUS data and 0.87 for the US data, respectively. The validation of the image fusion was limited to a subjective expert judgment. A similar image registration problem was taken up by [112], where the authors registered HFUS images of finger vascular tissue over time and measured its deformations under an ultrasound transducer.

Both the mentioned works [79,80] are summarized in Table 7.

#### Skin Vessel Segmentation Summary

The accurate segmentation and visualization of superficial veins are essential in diagnosing skin lesions and the most crucial findings in this field can be summarized as follows:

- Only two works targeted HFUS image segmentation with the highest accuracy reported by Mathai et al. [79] using the level-set technique.
- There appeared to be more algorithms concerning vessel segmentation using the classical US (<20 MHz) [113], which can also be applied to HFUS image data. In [80], the same active contour-based framework was utilized for ultrasound images acquired with different transducers (12 and 22 MHz) and other US machines.
- Accurate superficial vein segmentation can be utilized for inter-modality image registration.
- The developed approaches required manual seed-point selection and their repeatability in connection with this choice was not discussed in the papers.

### 4.4. Skin Lesion Classification

In addition to the segmentation methods, image classification techniques were among the most explored in the CAD systems. The developments in this area have made it possible to classify skin diseases, with the most significant emphasis being on skin tumors.

#### 4.4.1. Skin Tumors

The classification of HFUS images was first mentioned in the work [81] by Kia et al., where the authors focused on distinguishing between healthy and cancerous tissues and benign and malignant lesions, as well as categorizing basal cell carcinomas and melanomas. The AI-based framework started with pre-processing steps including color scale conversion and contrast enhancement. Next, the Canny edge detection step was applied, followed by morphological operations and ROI selection. The obtained regions were then used for the feature extraction and classification phases. The designed multilayer perceptron (MLP) network utilized the normalized mean squared error (MSE) as a transfer function. The evaluated database consisted of 120 images, resulting in a sensitivity of 98% and a specificity of 5%, which is clinically unacceptable. There was also a lack of information concerning the sizes of the individual groups. Additionally, the authors did not provide visual examples of the sample images, and it was hard to determine how complicated the classification problem was. Since the authors of [81] did not include information concerning the HFUS device, this field in Table 8, which summarizes the classification approaches, was completed based on our own experience.

The second work in this area by Csabai et al. [82] targeted distinguishing common skin lesions (BCC, melanoma, nevus). The three-step algorithm utilized a semi-automated active contour model to segment the lesion. However, the influence of the segmentation results on further classifications was not discussed. Next, which has been widely explored in the literature, the object features (both shape- and intensity-based) were calculated to describe the segmented area. The ROC curve enabled the selection of the most powerful features and classifiers (AdaBoost or support vector machine, SVM). The image data were collected using a HI VISION Preirus, Hitachi (Tokyo, Japan [36]), 5–18 MHz (EUP-L75 transducer). The obtained results were promising but, as the authors stated, were still unacceptable for clinical usage (see Table 8).

Melanocytic skin tumors were also an interest of Andrekute et al. [83]. The study involved 119 patients with malignant melanoma (MM), 48 cases, and benign melanocytic nevi (MN), 71 cases, confirmed histopathologically, from which 160 ultrasound datasets were acquired. The ROI segmentation algorithm utilized previous developments by the authors [110], and the tumor segmentation step applied parametric integrated backscattering signals, highlighting the different intensity object boundaries evaluated in [110]. The next step, the feature extraction step, included twenty-nine parameters (acoustical, textural, and shape), from which an informative set was selected and used in the SVM classifier. The obtained classification results are summarized in Table 8.

Three years later, Kia et al. [45] developed a skin image classification algorithm based on a discrete cosine transform (DCT), followed by a singular value decomposition (SVD) and neural network with two hidden layers (10 neurons each) and a CE loss function. The extended (compared to [81]) database of 400 HFUS samples included melanomas, basal cell carcinomas, squamous cell carcinomas, actinic keratosis, atypical nevi, benign melanocytic nevi, blue nevi, and seborrheic keratosis. However, the classification step was limited to four groups, as in [81], or two groups, healthy and suspected.

The skin lesion classification problem was also taken up by Tiwari et al. [46] (an extension of [110]), where the authors investigated the different classification models, logistic regression (LR), linear discriminant analysis (LDA), support vector machine, and naive Bayes, to analyze multimodal image data. The hybrid technique utilized the benefits of dermatoscopy, high-frequency ultrasound, and spectrometry in supporting the diagnosis of cutaneous melanoma differentiation between melanocytic naevus and melanoma. The analyzed dataset consisted of 50 nevus and 41 melanomas for which the three mentioned modalities were acquired. The classical framework included ROI selection, feature extraction, and a classification step. The experiments proved the improvement of classification performance by combining three imaging modalities compared to a combination of any two. The SVM classifier outperformed all the considered methods, resulting in an accuracy equal to 0.989 and an area under the ROC curve equal to 0.999.

The newest work by Marosán-Vilimszky et al. [21] was the last to focus on nevus. The analyzed HFUS recordings included 310 lesions comprising 70 melanomas, 130 basal cell carcinomas, and 110 benign nevi. The designed framework started with the fully-automated segmentation step described in [20] and the provided evaluation included a comparison of two semi-automated techniques, from which the best performance ensured the largest area rectangle-based technique (LAR). The rectangle created on the freehand drawing was used here as the seed. From the segmented regions and the surrounding tissues, the authors extracted 93 features (textural- and shape-based) from which they finally selected 62 for the SVM-based classification step. Compared to the similar work by Csabai et al. [82], the authors obtained [21] better classification results. It is worth mentioning that the authors shared all the code used in this work on GitHub [91]. Non-melanocytic skin tumors were also considered in [74]. However, in this work, the classification problem was broader and the authors focused on different skin lesions including tumors and inflammatory skin diseases. Thus, the analyzed dataset consisted of 631 HFUS images classified into four groups: non-melanocytic skin tumors, psoriasis, atopic dermatitis, and healthy skin. Since the most challenging task was the classification of inflammatory skin diseases, skin tumors were an additional group for the analysis and were not the target itself. The developed framework utilized deep neural networks and the authors considered the five different CNN models that offered the most promising effectiveness in the experiments. All the models were pre-trained on the ImageNet database [114]. The numerical evaluation utilized the ROI selection introduced as a pre-processing step and two augmentation methods. An exciting novelty of this work was the model evaluation section. The CNN model was selected by applying the Grad-CAD [115] algorithm and pre-segmented ROI skin layer map. The best model was this one as it resulted in the highest accuracy and focused on the same image area as the specialist. In this case, it was DenseNet-201 pre-trained on ImageNet, with augmentation (horizontal reflection and ±10-pixel translation in both directions), which was fed by the extracted ROI.

#### 4.4.2. Skin Burns

Although monitoring burn treatment and measuring its depth over time is still challenging, there is only one work targeting this problem described in the literature [86] that utilized the benefits of HFUS. The lack of these solutions may be related to the low penetration depth of HFUS not being appropriate for deep burn assessment. The presented approach enabled the real-time classification of burn depth in US images. The dataset of skin tissues with various degrees of burns was obtained from ex vivo porcine experiments using an Ultrasonix (USA) L40-8/12 linear array probe at 10 MHz (a frequency lower than in other skin applications) and included 143 images. The burns ranged from superficial-partial to full-thickness and were divided into four categories. The developed framework applied textural features for the SVM classifier and resulted in an overall classification accuracy equal to 0.93. The obtained results are included in Table 9 as a separate category that is highlighted in blue.

#### 4.4.3. Skin Lesion Classification Summary

Skin lesion classification was the second most explored research area in HFUS processing and the main findings can be summarized as follows:

- Two solutions prevailed in the analyses: SVM (with both textural and shape-based features) and neural networks, with a particular emphasis on classical multilayer perceptron networks.
- Since the datasets for the image classifications were less time-consuming to collect, the described experiments considered larger samples compared with the skin lesion segmentation experiments.
- Although all the authors outlined the potential of HFUS for dermatological diagnosis, the best-reported results [46] were obtained for multimodal analysis utilizing an HFUS (22 MHz), optical dermatoscopy, and a spectrophotometer. For 91 multimodal samples, the accuracy of the melanocytic lesion classification (malignant melanoma and melanocytic nevi) was equal to 0.989 and the AUC was close to one.
- For the analysis of single modality (400 images, 50 MHz), noteworthy results (AUC equal to 0.917) were reported by [45] in the four-group classification: healthy skin, benign nevi, BCC, and melanoma. For the two-group analysis (310 benign and malignant lesions), the best solution was provided in [21], with an AUC equal to 0.953.
- An extension of the skin tumor analysis was conducted by [74], which included non-melanocytic skin tumors in the differential diagnosis of different skin lesions.

### 4.5. Other Applications

Water loss is a particular problem that plays a vital role in many medical and beauty treatment applications [52]. The evaluation of water content was the area of interest of Chirikhina et al. [23,52]. Their first work [52], being less technical, was referred to in Section 3. The second one [23] presented a machine learning-based skin characterization method, which utilized a combination of contact capacitive and HFUS imaging for complementary information. The work aimed to measure skin water content and skin layer thickness in different skin sites (mainly facial). For the HFUS data acquisition, the authors used an EPISCAN I-200, Longport (Chadds Fort, PA, USA) system [34], with a 50 MHz transducer. Although the authors proposed a multimodal approach, the two mentioned modalities were analyzed separately. For the skin contact capacitive image classification, they utilized pre-trained deep neural network models, whereas the HFUS images were analyzed using different classifiers utilizing luminosity values. Another examined HFUS image classification method considered texture features and the DNN model as the particular feature selector. Unfortunately, the described findings were not supported by numerical analysis.

### 4.6. Image Quality Assessment

Apart from classical CAD algorithms, the two most recent works [55,89] on HFUS data analysis targeted the broadly defined image quality assessment (IQA). The main goal of IQA techniques is to reduce noise and artifacts, which influence the geometry of visualized structures and may lead to misclassification, false-positive detections, over/under-segmentation, and consequently, the inaccurate results of the measurements [89]. The issue of image artifacts and their effect on data quality in critically ill subjects were described in [24]. The outcome of this study was 1761 HFUS (20 MHz) images of 137 patients at risk of pressure ulcers, which were acquired and evaluated. At least one group of artifacts (bubbles, texture problems, layer non-differentiation, or reduced area of view) was found in 83% of images but they mainly did not interfere with the evaluation. The artifacts that the operator needs to recognize quickly during scanning because of their adverse effect on HFUS evaluation are layer non-differentiation, texture problems, and reduced area of view. Czajkowska et al. [89] added to this group of artifacts with the US probe or impurities contained in the ultrasound gel; frames captured when the ultrasound probe was not adhered or incorrectly adhered to the patient’s skin; images with contrast that was too low for a reliable diagnosis or captured with too little gel volume-improper for epidermis layer detection; and data with disturbed geometry, as well as HFUS frames with common ultrasound artifacts such as acoustic enhancement, acoustic shadowing, or beam-width artifacts.

Since in [24] the operator observed the scans as they were generated and saved the best for later analysis, most of the problems described in [89] did not appear in [24]. On the other hand, the framework described in [89] was fully automated and the selected good-quality frames were successfully used in CAD systems.

The latest works in medical IQA are based on deep neural networks [116,117,118] applied to retina images [116], heart MRI [118], or abdominal ultrasound [117]. A similar methodology was used in [55,89] for HFUS image classification.

The algorithm described in [55] targeted reducing the analyzed dataset of HFUS of hyaline cartilage at the metacarpal head level to the informative part. The B-scans were acquired using the 22 MHz transducer with a penetration depth of 15 mm. The authors introduced the CNN first for the HFUS data analysis. However, the application area was not connected with skin diseases and is not discussed in detail in our study. Briefly, the developed framework was evaluated on 48 healthy subjects, 40 in the training and testing of the algorithm and 8 in the reliability analysis, and the proposed method introduced the VGG16 [87] model to solve the classification problem.

The already mentioned framework [55] was, however, an inspiration for [89], where the authors focused on the HFUS image classification of healthy skin. The study’s goal was an automatic selection of the correct frames from the acquired image series in the CAD system. The IQA prevented further processing errors connected with inaccurate data analysis. The publicly available [88] dataset consisted of 17425 HFUS frames of the facial skin of 44 healthy volunteers denoted by two experts (in total, three times) as noisy-inaccurate, non-informative, and good quality. The authors evaluated different algorithms to classify them into two (as correct or incorrect) or four groups (including two, ‘almost’ correct and ’mostly’ incorrect). The numerical analysis included the VGG16 [87] model recommended in [55], as well as the DenseNet-201 [85] model, both pre-trained on ImageNet [114] and then used in transfer learning. Additionally, the authors proposed a novel framework connecting the CNN with fuzzy set theory (Mamdani Interference System, MIS [119]).

A critical issue connecting the two mentioned IQA works [55,89] is the expert annotation analysis preceding the main experiments. According to [120], the intra- and inter-observer agreements were interpreted using unweighted Cohen’s kappa [121] and confusion matrices.

The summary of IQA algorithms for facial HFUS [89] image processing is given in Table 10. It includes the range of the results obtained utilizing the referred methods and the information concerning the number of considered classes (two or four). Based on this information, the VGG16 model and its combinations with other machine learning methods are the most promising. However, since there are only two works described in the literature, the IQA of HFUS requires further investigation in the future.

## 5. Data Repositories

In the era of the continuous development of artificial intelligence methods, one of the main limitations is access to training data. The publicly available datasets containing labeled (for classification), annotated (for classification and detection), or manually delineated (for segmentation) images are the driving force for inventing new algorithms or enabling the training of already developed complex models. Although sharing data is an increasingly popular trend and publishers encourage this, only two HFUS image datasets are available in the public domain (see Table 11). Most of the referred papers described the repositories collected by the authors in [15,20,21,23,26].

The two shared datasets are [67,88]. Both are available with a CC BY 4.0 license in the Mendeley Data repository. The first one [67] consists of 380 HFUS images of different patients with AD (303) and psoriasis (77). The file names make it possible to distinguish between the types of visualized diseases. Therefore, they can be used as labels for further classification tasks. Yet, the authors did not include such information in the repository description. The data were acquired using a DUB SkinnScanner75, tpm (Lueneburg, Germany) [30] with a 75 MHz transducer. The images have the same size of 2067 × 1555 × 3 pix (RGB) with four different resolutions (lateral × axial): 0.0019 × 0.085, 0.0024 × 0.085, 0.0031 × 0.085, and 0.0019 × 0.085 mm/pix. Entry echo epidermis and SLEB layer expert delineations are provided for each image. However, the descriptions were from a single expert only. Additionally, the authors shared the SegUnet model developed in [3] that was pre-trained on the referred dataset for the epidermis and SLEB segmentation. Besides the work by [3], the set was also used in [17,48] in the experiments on HFUS image segmentation and partially in [74], which targeted HFUS data classification.

The second one [88] includes HFUS images of facial skin. The data were acquired using the same scanner (DUB SkinnScanner75, tpm (Lueneburg, Germany) [30]) but with a lower frequency transducer of 24MHz. The original size of the acquired images was 3466 × 1386 × 3, whereas due to the limited space, the shared image data are of the size 224 × 224 × 3. This corresponds to the input image size required by the applied CNN models [74]. However, the authors claimed that the actual data would be shared for individual requests. The images were collected during four sessions with 44 postmenopausal patients in 3 different facial locations. Each registered series includes the image data suitable for further diagnosis (technical, using CAD software, or medical) and non-informative images. Three experts described the images’ usefulness and the labels are included in the repository. Similar to the previous dataset, the repository contains CNN models trained to solve the classification task described in [89], that is, to divide the dataset into two groups (informative and non-informative).

The skin HFUS datasets are summarized in Table 11. It also has to be mentioned that according to the data availability statement currently required by most of the publishers, the authors of the most recent papers included it in their works [4,86]. However, as the authors stated, the data are available from the corresponding author upon reasonable request.

## 6. Conclusions and Future Direction

The paper summarizes CAD algorithms for dermatology, allergology, cosmetology, and aesthetic medicine, utilizing HFUS image data. The most frequently used solutions are discussed and the ones with potential for further development and clinical application are selected.

**Figure 5:**
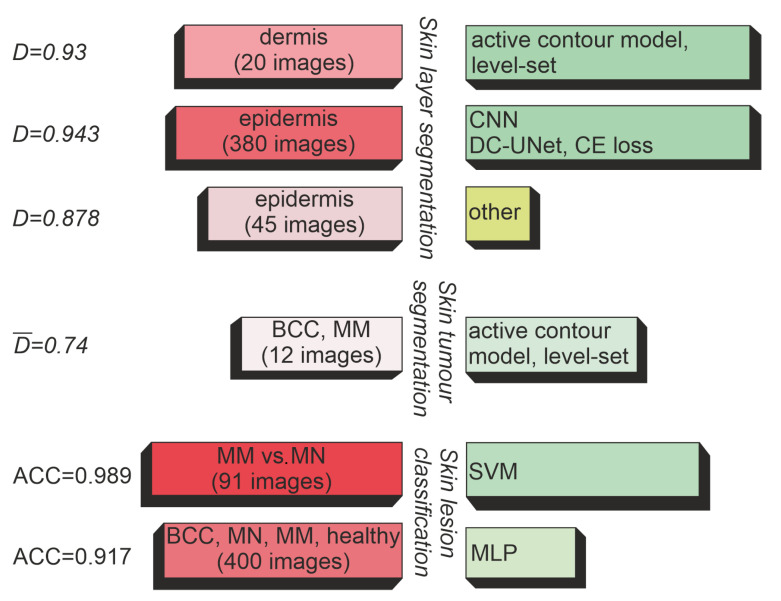
Summary of the most developed areas of CAD of skin diseases in HFUS.

Since 2005 when the first solution was published, plenty of work has appeared, mainly focusing on three groups. They include HFUS image segmentation, skin layers and skin tumors, and HFUS image classification, where the majority of methods focus on pigmented lesions (malignant melanomas, benign melanocytic nevi) or non-melanocytic nevi (such as BCC). The most practical approaches in these three groups and the highest obtained accuracies are summarized in Figure 5. The two methods that deserve special attention are epidermis segmentation based on DC-UNet [48] and image classification using multilayer perceptron networks [45], which were evaluated using numerous datasets, and the machine learning approaches presented in that study enable further training with new data samples.

In addition to those mentioned above, the described solutions include inflammatory skin disease assessment by image classification, where an early differentiation between psoriasis and atopic skin dermatitis is especially crucial [74]; subcutaneous vessel segmentation [79], which is applicable in further tracking and image registration methods; skin burns assessment [86]; and water content evaluation [23], which is important in cosmetology and aesthetic medicine.

There also appeared works targeting image quality assessment [55,89], a substantial pre-processing step in CAD systems, which complemented the previously mentioned works. Additionally, among the many other solutions, two deserve special attention [3,89] since they describe the publicly available data repositories [67,88].

According to all the descriptions and Tables included in this work, we can conclude that the CAD algorithms in the area of HFUS-based skin analysis have developed dynamically and have the potential to be robust and accurate in clinical practice. Yet, there is still room for development. As reported in [56], the fast development of CAD systems requires strong cooperation between clinical and technical experts. The clinical usages of HFUS are widely described in the literature and supported by clinical tests [4,40]. However, this modality is still not commonly used in medical practice. This results in few experts and clinics being able to prepare annotated or delineated image data for the further development of computerized methods. However, increasing interest in the medical community of HFUS for skin analysis, together with emerging solutions in automated HFUS image processing, should increase the number of datasets dedicated to CAD development and, consequently, the developed methods.

Based on our observations, the highest potential is for algorithms that utilize machine learning, and similar to other implementation areas, they should also be developed in this field. However, the already mentioned data access problem constitutes a severe limitation. In this case, semiautomatic techniques known from other segmentation problems can help (active contour models, level set, etc.).

The most crucial avenue for future work is the complex CAD solution, including image classification and segmentation, in each of the considered diagnostic fields: dermatology, dermatological oncology, and allergology. However, these three paths can be treated separately. The system should be able to diagnose skin diseases, that is, classify the skin lesion, estimate its size, and quantitatively and qualitatively describe it and the surrounding tissues. Therefore, an accurate skin layer and lesion segmentation algorithm should be the first of the tools developed. Unfortunately, the already described solutions only include selected layer or lesion analysis, therefore, they are limited to single applications.

Further development can start by utilizing the already implemented segmentation techniques that the HFUS machine developers provided. Both the DUB SkinnScanner75, tpm (Lueneburg, Germany) [30] and the Dermascan C, Cortex (Aalborg, Denmark) [31] enable automated skin layer segmentation (epidermis and dermis). The study by Alsing et al. [122] compared the manual and automatic skin layer segmentation provided by the Dermascan C with manual expert delineations. The results differed by 20% and the results’ quality was worse than that described in the literature for CAD methods. However, none of the cited segmentation methodology authors compared their results with existing ones. Moreover, these results can be used as a pre-processing step for further automated analysis.

The problems with access to the HFUS image data have resulted in the over-adaptation of the algorithm to one device. All the described methods were evaluated using a single machine and are not universal solutions. This is at odds with the recently promoted idea of cloud systems, where the image data are provided irrespective of their origins. It is now possible to utilize CT or MRI datasets, for which many CAD solutions have been described in the literature and implemented commercially. HFUS data exchange between scientific centers and publishing databases could significantly speed up the development of solutions for their analysis.

The analysis of many modalities also has excellent potential, as confirmed in [46]. Methods utilizing dermatoscopy and HFUS in a single diagnosis will provide the user with a complete diagnostic picture and should be the next direction to take for future works.

To sum up this survey, we can suggest a few questions and open problems that were not discussed in the reviewed papers, however, they need to be addressed in future works in this area:

- Considering the differences in multiple manual delineations, what is the segmentation accuracy and size of the validating dataset that confirms the clinical acceptance of the automated processing method?
- Assuming that for HFUS image data, the acquisition protocol and the way of data collection influence the registered images, is it possible to design a segmentation or classification algorithm that results in similar values (segmentation masks or classification results) for the same patient examined by different physicians? What is an acceptable level of hypothetical difference?
- Is it possible to create a universal system that is able to process HFUS images from different clinical applications and is there such a need at all?
- Is it possible to precisely define the current and most crucial needs for CAD methods in HFUS image analysis?

## Figures and Tables

**Table 1:** HFUS machines available on the market and their acquisition parameters.

Product	Frequencies (MHz)	Penetration Depth (mm)	Max. Axial Resolution (μm)
tpm (Lueneburg, Germany) [30], DUB 100-12 bit	75, 100	3.2, 1.6	21, 16
tpm (Lueneburg, Germany) [30], DUB SkinScanner75	18, 22, 33, 50, 75	15, 10, 6, 4, 3	72, 57, 42, 31, 21
Cortex Technology (Aalborg, Denmark) [31], Dermascan C	20, 20 (long), 50	15, 23, 3	60, 60, 30
FUJIFILM VisualSonics (Toronto, ON, Canada) [32], Vevo 3100, (MX700 transducer)	29–71	up to 36	down to 30
Dramiński (Gietrzwałd, Poland) [33], DermaMed	48	up to 5	16
Longport Inc. (Chadds Fort, PA, USA) [34], Episcan I-200	50	3.8, 5.4, 7.6, 11.2, 15, 22.4	-
Atys Medical (Soucieu en Jarrest, France) [35], Atys Dermcup	16–50	up to 12	down to 30
Hitachi (Tokyo, Japan) [36], HI VISION Preirus (EUP-L75 transducer)	5–18	-	-

**Table 2:** Clinical applications of HFUS with dedicated CAD methods.

Application Area	Work	Description	HFUS Device	Database	CAD
Dermatological oncology	Wang et al. [44]	assessment of the ultrasonographic features of BCC (differentiation)	MD300S II, MEDA Co. (Tianjin, China), 20 and 50 MH	42 patients, 46 lesions: 6 high-risk BCC, 40 low-risk BCC	Sciolla et al. [26], skin tumor segmentation, Marosán-Vilimszky et al. [20], Kia et al. [45] skin lesion classification
	Reginelli et al. [28]	role of HFUS for the nodular skin melanoma Breslow thickness in adults before surgery by making a comparison with histological features	Vevo, FUJIFILM Visual Sonics [32] (Toronto, ON, Canada), 70 MHz	14 melanocytic lesions	Sciolla et al. [26], skin tumor segmentation, Tiwari et al. [46], skin lesion classification
Dermatology and allergology	Polańska et al. [47]	comparison of high-frequency ultrasonography and histopathology in atopic dermatitis, skin echogenicity, and thickness analysis	Dermascan C, Cortex [31] (Aalborg, Denmark), 20 MHz	16 patients suffering from AD and 15 healthy individuals without any signs of atopic or chronic diseases	Sciolla et al. [10], Czajkowska et al. [15], Szymańska et al. [48], epidermis, dermis, and SLEB segmentation
	Polańska et al. [49]	calcipotriol/betamethasone ointment compared to narrow-band UVB in plaque psoriasis, measurement of SLEB thickness as an objective parameter to assess skin lesions	Dermascan C, Cortex [31] (Aalborg, Denmark), 20 MHz	58 consecutive patients diagnosed with recurrent chronic small plaque psoriasis	Czajkowska et al. [15], Szymańska et al. [48], epidermis, dermis and SLEB segmentation
Aesthetic medicine and cosmetic applications	Crisan et al. [50]	cutaneous changes induced by topical use of a vitamin C complex at facial level	Dermascan C, Cortex [31] (Aalborg, Denmark), 20 MHz	60 Caucasian female healthy individuals, aged between 20 and 75 years	Sciolla et al. [10], Czajkowska et al. [15], Szymańska et al. [48], epidermis and dermis segmentation
	Meng et al. [51]	facial skin thickness in association with gender, age, and BMI	Aplio i800, Canon Medical Systems (Tustin, CA, USA), 24 MHz	118 healthy adults: forehead, glabella, temple, eyelid, nasal dorsum, zygoma, submandibular, and neck	Sciolla et al. [10], Czajkowska et al. [15], Szymańska et al. [48], epidermis and dermis segmentation
	Chirikina et al. [52]	water content, transepidermal water loss (TEWL), and thickness in facial skin	Episcan I-200, Longport (Chadds Fort, PA, USA), 18, 35 and 45 MHz	48 healthy patients, 6 regions: cheek, chin, forehead, lips, neck, nose	Sciolla et al. [10], Czajkowska et al. [15], Szymańska et al. [48], epidermis and dermis segmentation
Other	Jain et al. [53]	evaluation of skin and subcutaneous tissue thickness at insulin injection sites	11 MHz	101 patients with insulin naive type 2 diabetes: upper arm, upper thigh, abdomen	Sciolla et al. [10] epidermis and dermis segmentation
	Gutierrez et al. [54]	inter-observer reliability of high-resolution ultrasonography in the assessment of bone erosions in patients with rheumatoid arthritis (RA): experience of an intensive dedicated training programme	MyLab 70 XVG, Esaote Biomedica (Genova, Italy), 6–18 MHz	20 consecutive patients with a diagnosis of RA	Cipolletta et al. [55] ultrasound informative image selection of metacarpal head cartilage

**Table 3:** Colour nomenclature.

Color	Description
	The most accurate among all the solutions in this category and evaluated using numerous datasets
	The most or second most accurate among all the solutions in this category and evaluated using numerous datasets
	The second or third most accurate in this category and evaluated using numerous datasets
	An interesting work worth mentioning
	An interesting work worth mentioning with different application fields

**Table 4:** Summary of skin layer segmentation algorithms for small, specific datasets.

Authors/Implementation	Algorithm	Segmented Area	HFUS Device	Database	Results
Lagarde et al. [27]	active contour model	dermis	Atys Medical (Soucieu en Jarrest, France) [35], Atys Dermcup, 20 MHz	612 images, different body parts	correlation with manual measurements $R^{2}=<0.0086,0.6097>$, semi-manual measurements $R^{2}=<0.6575,0.9406>$
	**Contribution**: the first solution in the HFUS image segmentation area; standardizing the manual dermal thickness measurement procedure; large database; fully automated; repeatable; **Disadvantages**: outperformed by further modifications to active contour model; single imaging device				
Gao et al. [64]	active contour model	dermis	Ultrasonix, Sonix RP (Richmond, BC, Canada), 10 MHz	730 images of breast skin, 23 patients	correlation with manual measurements $R^{2}=<0.7,0.74>$, difference of skin thickness in comparison with both experts <5%
	**Contribution:** new automated dual-curve evolution technique; large database; fully automated; repeatable; **Disadvantages:** low transducer frequency, not typical for skin diagnosis; outperformed by further modifications to active contour model; single imaging device				
Sciolla et al. [10]	active contour model, level set	dermis (epidermis, SLEB-not evaluated)	Atys Medical (Soucieu en Jarrest, France) [35], Atys Dermcup, 50 MHz	20 images, left forearm	median(MAD) = 45μm, mean(MAD) = 62±46μm, median(D) = 0.94, mean(D) = 0.93±0.055
	**Contribution:** new non-parametric active contour method; texture criterion combined with the geometric constraint; the highest accuracy among all the classical solutions; **Disadvantages**: small database; single imaging device; moderate numerical costs; the contour initialization method was not discussed				
Gao et al. [64]/Sciolla et al. [10]	active contour model	dermis	Atys Medical (Soucieu en Jarrest, France) [35], Atys Dermcup, 50 MHz	20 images, left forearm	median(MAD) = 65 μm, mean(MAD) = 99 ± 99 μm, median(D) = 0.93, mean(D) = 0.82±0.28
	**Contribution:** implementation of previous (state-of-the-art) solution [64] to new data as a reference				
Chan-Vese et al. [65]/ Sciolla et al. [10]	active contour model	dermis	Atys Medical (Soucieu en Jarrest, France) [35], Atys Dermcup, 50 MHz	20 images, left forearm	median(MAD) = 150 μm, mean(MAD) = 160± 65 μm, median(D) = 0.9, mean(D) = 0.9±0.046
	**Contribution:** implementation of the state-of-the-art method [65] to new problem as a reference				
Czajkowska et al. [14]	FCM clustering	epidermis	DUB SkinnScanner75, tpm (Lueneburg, Germany) [30], 22 MHz	13 images	upper epidermis boundary: mean (HD) = 118± 48 μm, lower epidermis boundary: mean(HD) = 145± 40 μm, mean(D) = 0.848±0.044
	**Contribution:** first method for epidermis segmentation; fully automated; repeatable; **Disadvantages:** preliminary work; limited dataset; single imaging device;				
Czajkowska et al. [15]	level set combined with FCM clustering	epidermis, SLEB	DUB SkinnScanner75, tpm (Lueneburg, Germany) [30], 75 MHz	45 images, different body parts, 45 patients	upper epidermis boundary: median(MAD) = 8.5 μm, difference in skin thickness in comparison with both experts <4%, median(D) = 0.878, mean(D) = 0.878±0.041
	**Contribution:** new approach combining FCM and level-set techniques; first method for SLEB segmentation; bigger dataset in comparison with [10]; high accuracy; fully automated; repeatable; **Disadvantages:** single imaging device; single disease;				
Czajkowska et al. [16]	U-Net	epidermis, SLEB	DUB SkinnScanner75, tpm (Lueneburg, Germany) [30], 75 MHz	47 images, different body parts, 47 patients	SLEB segmentation: mean(D) = 0.86±0.04, mean(MAD) = 12± 9.3 μm
	**Contribution:** first method utilizing CNN for skin layer segmentation; fully automated method; repeatable; **Disadvantages:** lower accuracy compared to previous method; small dataset for deep learning; single disease				

**Table 5:** Deep learning-based approaches for skin layer segmentation in HFUS.

Authors of the Algorithm/ Implementation	Algorithm/ Optimizer	Segmented Area	HFUS Device/Input Image Size	Database	Results
Ronneberger et al. [66]/ Czajkowska et al. [3]	U-Net, CE loss, SGDM	epidermis, SLEB	DUB SkinScanner75 [30] 75 MHz/128×64	380 images [67]: 303 AD, 77 psoriasis	epidermis: median(D) = 0.869, SLEB: median(D) = 0.815
	**Contribution:** application of state-of-the-art CNN model (in medical image processing) to HFUS data as a reference				
Badrinarayanan et al. [68]/ Czajkowska et al. [3]	SegNet, CE loss, SGDM	epidermis, SLEB	DUB SkinnScanner75, tpm (Lueneburg, Germany) [30] 75 MHz/128×64	380 images [67]: 303 AD, 77 psoriasis	epidermis: median(D) = 0.737, SLEB: median(D) = 0.463
	**Contribution:** application of state-of-the-art CNN model (in medical image processing) to HFUS data as a reference				
Gu et al. [69]/ Czajkowska et al. [3]	Ce-Net, CE loss, SGDM	epidermis, SLEB	DUB SkinnScanner75, tpm (Lueneburg, Germany) [30] 75 MHz/128×64	380 images [67]: 303 AD, 77 psoriasis	epidermis: median(D) = 0.867, SLEB: median(D) = 0.798
	**Contribution:** application of state-of-the-art CNN model (in medical image processing) to HFUS data as a reference				
Czajkowska et al. [3]	SegUnet, CE loss, SGDM	epidermis, SLEB	DUB SkinnScanner75, tpm (Lueneburg, Germany) [30] 75 MHz/128×64	380 images [67]: 303 AD, 77 psoriasis	epidermis: median(D) = 0.874, SLEB: median(D) = 0.829
	**Contribution:** new DNN model utilizing the advantages of U-Net and SegNet model; fully automated; repeatable; the pre-trained CNN models are publicly available; **Disadvantages:** outperformed by later solutions; limited diseases; single imaging device				
Siddique et al. [70]/ Szymańska et al. [48]	U-Net, CE loss, Adam	epidermis, SLEB	DUB SkinnScanner75, tpm (Lueneburg, Germany) [30] 75 MHz/512×256	380 images [67]: 303 AD, 77 psoriasis	epidermis: median(D) = 0.927, SLEB: median(D) = 0.905
	**Contribution:** application of the most recent CNN model to HFUS data as a reference				
Lou et al. [71]/ Szymańska et al. [48]	DC-UNet, CE loss, Adam	epidermis, SLEB	DUB SkinnScanner75, tpm (Lueneburg, Germany) [30] 75 MHz/512×256	380 images [67]: 303 AD, 77 psoriasis	epidermis: median(D) = 0.943, SLEB: median(D) = 0.930
	**Contribution:** application of the most recent CNN model to HFUS data; the highest accuracy among all the HFUS image segmentation techniques; fully automated method; repeatable; **Disadvantages:** limited diseases; single imaging device				
Lou et al. [72]/Szymańska et al. [48]	CFPNet-M, CE loss, Adam	epidermis, SLEB	DUB SkinnScanner75, tpm (Lueneburg, Germany) [30] 75 MHz/512×256	380 images [67]: 303 AD, 77 psoriasis	epidermis: median(D) = 0.932, SLEB: median(D) = 0.915
	**Contribution:** application of the most recent CNN model to HFUS data; the second highest accuracy among all the HFUS image segmentation techniques; fully automated; repeatable; **Disadvantages:** limited diseases; single imaging device				
Chen et. al. [73]/ Czajkowska et al. [74]	DeepLab v3+, Xception, Dice loss	epidermis	DUB SkinnScanner75, tpm (Lueneburg, Germany) [30], 75 MHz/224×224	580 images: 303 AD [67], 77 psoriasis [67], 200 tumors	epidermis: median(D) = 0.899
	**Contribution:** application of CNN model originated for layered image analysis to HFUS data; extended database including various diseases; fully automated; repeatable; **Disadvantages:** outperformed by later solutions; the analysis is limited to the epidermis; single imaging device				
Czajkowska et al. [17]	DeepLab v3+, ResNet-50 +FC, Dice loss	epidermis	DUB SkinnScanner75, tpm (Lueneburg, Germany) [30], 75 MHz/224×224	380 images [67]: 303 AD, 77 psoriasis	epidermis: median(D) = 0.930
	**Contribution:** application of state-of-the-art model in image segmentation to HFUS data; high accuracy; fully automated; repeatable; **Disadvantages:** outperformed by other solutions; the analysis is limited to the epidermis; single imaging device				
Czajkowska et al. [17]	DeepLab v3+, ResNet-50 +FC, Dice loss	epidermis	DUB SkinnScanner75, tpm (Lueneburg, Germany) [30], 75 MHz/224×224	580 images: 303 AD [67], 77 psoriasis [67], 200 tumors	epidermis: median(D) = 0.919, AD −> median(D) = 0.933, psoriasis −> median(D) = 0.937, tumor −> median(D) = 0.850
	**Contribution:** application of state-of-the-art model in image segmentation to HFUS data; high accuracy; extended database including various diseases; fully automated; repeatable; **Disadvantages:** outperformed by other solutions; the analysis is limited to the epidermis; single imaging device				

**Table 6:** Skin tumor segmentation summary.

Authors of the Algorithm/ Implementation	Algorithm	HFUS Device	Nb. of Cases/Lesion Type	Results
Pereyra et al. [75]	spatially coherent generalized Rayleigh mixture model	synthetic data/Atys Medical (Soucieu en Jarrest, France) [35], Atys Dermcup, 25 MHz, 3D reconstruction	30 slices, 2 lesions/phantom	only visual analysis provided
	**Contribution:** first approach to skin tumor segmentation in HFUS; synthetic and clinical data; fully automated; repeatable; **Disadvantages:** lack of quantitative analysis; highly limited dataset			
Sciolla et al. [76]	adaptive log-likelihood level set	Atys Medical (Soucieu en Jarrest, France) [35], Atys Dermcup, 50 MHz, 3D reconstruction	8 lesions, 13 phantoms	mean(D) = 0.756, mean(MAD) = 184 μm
**Contribution:** new adaptive level-set approach; synthetic and clinical data; quite high accuracy				
Chan and Vese [65] and Sarti et al. [77]/ Sciolla et al. [76]	non-adaptive log-likelihood level set	Atys Medical (Soucieu en Jarrest, France) [35], Atys Dermcup, 50 MHz, 3D reconstruction	8 lesions, 13 phantoms	mean(D) = 0.732, mean(MAD) = 177 μm
	**Contribution:** application of state-of-the-art method of medical image segmentation for HFUS data analysis as a reference			
Sciolla et al. [26]	hybrid geodesic active contour with Probabilistic Boundary Expansion term	Atys Medical (Soucieu en Jarrest, France) [35], Atys Dermcup, 50 MHz, 3D reconstruction	12 lesions: 3 BCC, 9 melanomas	mean(D) = 0.78±0.1, mean(MAD) = 200±110 μm
	**Contribution:** new hybrid active contour model for skin lesion segmentation; the highest accuracy among all solutions; **Disadvantages:** limited dataset; single imaging device; manual seed-point selection; lack of repeatability analysis			
Sciolla et al. [76]/ Sciolla et al. [26]	adaptive log-likelihood level-set	Atys Medical (Soucieu en Jarrest, France) [35], Atys Dermcup, 50 MHz, 3D reconstruction	12 lesions: 3 BCC, 9 melanomas	mean(D) = 0.74±0.1, mean(MAD) = 240±140 μm
**Contribution:** application of previous solution to extended dataset as a reference; quite high accuracy				
Qiu et al. [78]/ Sciolla et al. [26]	geometric active contour	Atys Medical (Soucieu en Jarrest, France) [35], Atys Dermcup, 50 MHz, 3D reconstruction	12 lesions: 3 BCC, 9 melanomas	mean(D) = 0.70±0.15, mean(MAD) = 330±190 μm
	**Contribution:** application of previous solution to extended dataset as a reference			

## Data Availability

Data sharing not applicable.
